# Notes and Comments

**Published:** 1893-08

**Authors:** 


### Notes and Comments.[1]

Pyorrhœa Alveolaris.—Many who have spoken at dental meetings upon this subject, and some who have written for the journals, have laid the cause to certain systemic conditions, such as gout and rheumatism; others attribute it to the excessive use of saline foods, etc.; while one writer says, "I have never seen an actual case of pyorrhœa alveolaris where the patient was in perfect health."

This subject is one deserving of greater attention and study than it apparently receives. Though we occasionally have valuable contributions, it seems difficult to bring the profession to one opinion

[1] The assistant editor solicits contributions for this department,—new methods, new remedies and formulas, or any short practical note which may prove of value to the practitioner or student. Address 1506 Arch Street, Philadelphia.

in regard to this branch of pathology. It can be stated, beyond a doubt, I think, that the appearance of pyorrhœa alveolaris is due to a calcic diathesis, as are a number of the dental lesions, such as hypercementosis and nodular dentine. This condition manifests itself by the deposit of calculus upon the necks and roots of the teeth, which deposit is either salivary or sanguinary, or both. In studying this subject, it must be remembered that salivary calculus causes inflammation, and that serumal or sanguinary calculus, which is found upon the roots of the teeth, above the gum margin, is the *result* of inflammation; and our observation leads us to the conclusion that this deposit is very often, if not nearly always, caused by the presence of the former, owing to its irritating effect resulting in inflammation of the soft tissue surrounding the teeth. While this, when promptly and properly treated, is not serious, it is, where the patient does not give careful and regular attention to the teeth, owing to its insidious character, one of the gravest diseases of the teeth, and is probably causing the loss of as many teeth as does caries.

After a tooth has been lost by this disease, examine it closely under a strong glass, and see how the pericemental membrane has been destroyed, and how the root is nearly, or quite, covered with a calcic deposit. Go farther: cut the tooth open, and see how the pulp has diminished in size, or has become calcified altogether. What does this mean, but simply that the patient is of a calcic diathesis? We wish to add, further, that during the past six months we have treated five cases of pyorrhœa alveolaris where the patients' general health was excellent.

---

THE FUNCTION OF THE SCHOOLS.—It has been charged frequently that the dental schools are engaged in making scientific men rather than dentists,—that is, that too much theory and not enough of the practical is taught. Instances are cited where graduates are not able to perform as good an operation as some practitioners who have never attended college, etc. In fact, one old practitioner, whose name is familiar throughout the country, made the statement, not many months ago, that he could take a young man into his laboratory and make a better dentist of him in six weeks than is the average college graduate. It might be said here that this same doctor (?) has recently contributed a series of papers to one of our professional journals which have to many been a sad disappointment, giving evidence of the absence of a good college training.

These criticisms, as Dr. Barrett says in the *Dental Practitioner and Advertiser*, are almost invariably made by illiterate men. He says further, and wisely, too, that the education of the dentist should not begin in the laboratory or operating-room. Before the student is prepared to commence operations in the mouth, he should be taught the underlying principles upon which a true practice must be founded. To put even the finest filling into a diseased tooth, perhaps over a dead and putrefying pulp, is not dentistry. To insert even the most beautiful artificial denture over tissues that are in an inflamed and sloughing condition is not good practice. All true practice must be founded upon true science. There must first be a comprehension of what is physiological law before one is fitted to deal with pathological cases.

---

Educating the Public.—Upon the subject of the dental education of the public, Dr. W. C. Barrett says, "I cannot but believe that the only dignified, professional, manly way is to continue within professional lines, keep out of the newspapers, and leave the advertising business to those who know more of it than we do; to diffuse information that is legitimate, whenever we have the opportunity to do so, in a place in which we know that it will be respected, and thus in time surely to build up a reputation that cannot be successfully attacked, and which will secure the respect even of the modern newspaper reporter."

Dr. Barrett usually says the right thing in the right place, and in calling attention to the above paragraph we can but add we have always felt that the best way to reach the public and educate them in dental matters is to elevate and broaden the education of those who enter the profession. What a responsibility for our colleges, examining boards, societies, and journals!

In fact, dentistry, as we have previously stated in these pages, seems to form a connecting link between the professions,—the healing art and mechanics,—and in its highest development exemplifies each.

